# Study on Optimal Broadcast Ephemeris Parameters for GEO/IGSO Navigation Satellites

**DOI:** 10.3390/s20226544

**Published:** 2020-11-16

**Authors:** Jin Haeng Choi, Gimin Kim, Deok Won Lim, Chandeok Park

**Affiliations:** 1 Department of Astronomy, Yonsei University, Seoul 03722, Korea; jhchoi1006@yonsei.ac.kr (J.H.C.); orion12@yonsei.ac.kr (G.K.); 2 GNSS R&D Division, Korea Aerospace Research Institute, Daejeon 34133, Korea; dwlim@kari.re.kr

**Keywords:** BeiDou, broadcast ephemeris parameters, least-square curve fit, nonsingular elements

## Abstract

This paper proposes new sets of suitable broadcast ephemeris parameters for geosynchronous (GEO) and inclined geosynchronous (IGSO) navigation satellites (NSs). Despite the increasing number of GEO and IGSO NSs, global positioning system (GPS)-type ephemeris parameters are still widely used for them. In an effort to provide higher fit accuracy, we analyze a variety of broadcast ephemeris parameters for GEO and IGSO satellites along with their orbital characteristics and propose optimal sets of parameters. Nonsingular elements and orbital plane rotation are adopted for alleviating/avoiding the singularity issues of GEO satellites. On the basis of 16 parameters of GPS LNAV, we add one to four parameters out of 28 correction ones to determine optimal sets of ephemeris parameters providing higher accuracy. All possible parameter sets are tested with the least-square curve fit for four BeiDou GEOs and six BeiDou IGSOs. Their fit accuracies are compared to determine the optimal broadcast ephemeris parameters that provide minimum fit errors. The set of optimal ephemeris parameters depends on the type of orbit. User range error (URE) accuracies of the proposed optimal ephemeris parameters ensure results within 2.4 cm for IGSO and 3.8 cm for GEO NSs. Moreover, the experimental results present common parameter sets for both IGSO and GEO for compatibility and uniformity. Compared with four conventional/well-known sets of ephemeris parameters for BeiDou, our proposed parameters can enhance accuracies of up to 34.5% in terms of URE. We also apply the proposed optimal parameter sets to one GEO and three IGSO satellites of QZSS. The effects of fitting intervals, number of parameters, total bits, and orbit types on the fit accuracy are addressed in detail.

## 1. Introduction

The navigation message, which is the final form of navigation data received by users, provides the location of navigation satellites (NSs). Users conduct navigation and positioning on the basis of the satellite location obtained from this message. As satellite locations are given in the form of ephemerides parameters for fitting, their fit performance directly influences navigation and positioning accuracy [1,2,3]. Hence, it is significantly important to design a set of appropriate/optimal broadcast ephemeris parameters in the navigation satellite system.

There exist two types of broadcast ephemeris parameters in general: Cartesian-based and Keplerian-based models. The Cartesian-based model describes the perturbed satellite orbit with instantaneous satellite position/velocity and lunisolar acceleration in the Earth-centered Earth-fixed (ECEF) coordinate system. It effectively estimates the position and velocity of satellites without singularity. However, a numerical integrator required to compute ephemeris increases computational time/cost [4]. The Keplerian-based model, on the other hand, describes the perturbed satellite orbit with Keplerian orbital elements and harmonic coefficients. It is significantly useful to represent the short arc of the perturbed satellite movement. However, the Keplerian-based model suffers from singularities; when the inclination or the eccentricity is close to zero value, the right ascension of the ascending node or the argument of perigee becomes unstable to cause inaccurate prediction of orbital ephemeris even for small perturbations [5,6,7]. The global positioning system (GPS)-type broadcast ephemeris, represented by the Keplerian-based model, is widely used in Galileo, BeiDou, QZSS, and IRNSS. However, the BeiDou, QZSS, and IRNSS constellations include inclined geosynchronous (IGSO) and geosynchronous (GEO) satellites which have clearly different orbital characteristics from those of GPS satellites [8,9,10,11,12,13]. Compared to MEO satellites of GPS, IGSO and GEO satellites have long orbital periods and are affected more by solar radiation pressure and third-body effects. Thus, the orbital elements and harmonic terms of GPS-type ephemeris parameters may be adjusted/improved for maximizing the fit performance for IGSO and GEO satellites. This motivates us to design a suitable set of ephemeris parameters for IGSO and GEO NSs by reflecting their orbital/constellation characteristics.

A few sets of broadcast ephemerides parameters have been presented to alleviate the singularity issues of Keplerian-type model and improve fit accuracy. Fu and Wu [14] introduced a set of optimal broadcast ephemeris parameters for IGSO, GEO, and MEO satellites. In this optimal parameter set, the singularity caused by small inclination of GEO satellites is resolved in such a way that their orbital reference planes rotate with a prescribed inclination angle. Xie et al. [15] designed a set of optimal broadcast ephemeris parameters for LEO satellites. They introduced nonsingular orbital elements suitable for LEO satellites by adding a few parameters to the GPS-type ephemeris. They presented fit accuracy for different parameter selection in the proposed ephemeris model. Du et al. [16] presented 18 elements of broadcast ephemeris parameters for GEO satellites, which is free from singularity even in small inclination by adopting nonsingular orbital elements. Furthermore, this ephemeris can precisely describe predominant characteristics of east–west drifts of the subsatellite point of GEO satellites by simply adding two parameters. Xiaogang and Mingquan [17] presented a 14-element broadcast ephemeris model for GEO, IGSO, and MEO satellites meeting the requirements of BeiDou. As this model can avoid singularities in small inclination/eccentricity by introducing nonsingular orbital elements, its algorithm for computing satellite positions is more complicated than that for the GPS-type ephemeris model.

This study presents a new set of broadcast ephemeris parameters minimizing user range error (URE) for IGSO and GEO satellites. To mollify the singularity issues caused by low inclination/eccentricity of IGSO/GEO orbits, nonsingular elements and the rotation technique of the orbital reference plane were adopted. The variations of orbital elements of IGSO and GEO satellites were analyzed. Then, the dominant perturbation forces and orbital variations were considered in establishing optimal ephemeris parameters. Feasible ephemeris parameter sets were generated by adding one to four parameters to GPS LNAV. The least-square curve fit was performed for optimal parameter selection in terms of fit accuracy and orbit type. Our proposed parameter sets for IGSO and GEO were compared with four representative ephemerides for fit accuracy. Consequently, the root mean square (RMS) of UREs for the proposed ephemerides achieved 1.92 cm for the 17-parameter set and 1.34 cm for the 20-parameter set, which were, respectively, 6.27% and 34.5% lower than those of well-known BeiDou ephemeris.

Some main contributions lie in this study. First, the core of the user algorithm for GPS-type ephemeris could remain essentially unchanged. The proposed sets of ephemeris parameters were distinctively designed by adding a few parameters to GPS LNAV ephemeris, which led to fit accuracy enhancement. User algorithms using GPS LNAV were not necessarily reconstructed for the proposed ephemeris parameter set. This idea was motivated by Xie et al. [15] and was carefully applied to the establishment/selection of compulsory and optional parameters in the design of the optimal ephemeris parameters for IGSO/GEO NSs. Second, we investigated a wide range of correction terms and orbital elements to establish the best parameter sets for IGSO/GEO satellites. The pool of additional parameters was set up to consist of 28 parameters, including the first/second/third order of periodic correction terms to four orbital elements and the first/second order of linear correction terms to six orbital elements. Through testing these various additional parameters, the optimal ephemerides for IGSO, GEO, and their combinations were comprehensively searched. Third, unlike some previous studies using only one specific satellite for validation, our proposed ephemeris parameters were tested via 10 BeiDou and four QZSS satellites to ensure reliability and adaptability for IGSO/GEO satellites. In addition, the relationship between total bits of the proposed ephemeris parameters and range error due to truncation (RET) was presented to investigate bit increments with parameter additions. The analysis/results can be useful in determining a reasonable set of ephemeris parameters, taking into account bit allocation and RET for designing the navigation message.

The remainder of this study is organized as follows: Section 2 describes the design of the proposed broadcast ephemeris parameter sets in detail. Section 3 shows the fit accuracy of the proposed ephemeris parameters according to parameter selections, the number of parameters, total bits, and fit interval. Comparison results with other ephemerides model are also presented. Section 4 draws a conclusion.

## 2. Design of Broadcast Ephemeris Parameters

This section presents the proposed ephemeris in detail. Keplerian elements have been used to describe orbital motions around the Earth. They are mainly subject to the central gravity but are also affected by perturbations such as spherical harmonics, the attraction of third bodies, atmospheric drag, solar radiation pressure, and Earth/ocean tide. These perturbations cause secular and periodic variations in the Keplerian elements [18]. The combination of Hamel and orthogonal basis can approximate these continuous variations to the desired accuracy as follows:

(1) $$\ell \left(t\right)=\ell_{L}\left(t\right)+\ell_{P}\left(t\right)+\delta ,$$

(2) $$\ell_{L}\left(t\right)=\varsigma_{0}+\varsigma_{1}t+\varsigma_{2}t^{2}+\varsigma_{3}t^{3}+\cdots ,$$

(3) $$\ell_{P}\left(t\right)=\xi_{1c}cos(\omega t)+\xi_{1s}sin(\omega t)+\xi_{2c}cos(2\omega t)+\xi_{2s}sin(2\omega t)+\cdots ,$$

where $\ell_{L}\left(t\right)$ denotes secular variation represented as the Hamel basis, $\ell_{P}\left(t\right)$ denotes periodic variation represented as an orthogonal basis, and δ denotes the remaining errors. The pair of (ω,t) denotes angular frequency and time. $\varsigma_{0},\varsigma_{1},\varsigma_{2},\varsigma_{3},\ldots$ and $\xi_{1c},\xi_{1s},\xi_{2c},\xi_{2s},\ldots$ are coefficients to be determined such that their optimal combination should minimize errors.

### 2.1. Orbital Characteristics of GEO and IGSO Satellites

The IGSO/GEO satellites are predominately influenced by spherical harmonics, attraction of third bodies (Sun and Moon), and solar radiation pressure in addition to central gravity [19]. Keplerian orbital elements (a,ecc,i,Ω,ω,M), which denote semimajor axis, eccentricity, inclination, right ascension of the ascending node (RAAN), argument of perigee, and mean anomaly, can typically express satellite motion around the Earth. However, Keplerian orbital elements have limitations in expressing near-circular or near-equatorial orbit due to singularity [6]. In an effort to resolve this singularity issue for near-circular orbit, we use nonsingular elements ($a,e_{x},e_{y},i,\Omega ,\lambda$), where

(4) $$e_{x}=ecccos(\omega ),e_{y}=eccsin(\omega ),\lambda =\omega +M.$$

These nonsingular elements can simply be converted to Kepler elements as

(5) $$ecc=\sqrt{e_{x}^{2}+e_{y}^{2}},\omega ={tan}^{-1}(e_{y}/e_{x}),M=\lambda -\omega .$$

The near-equatorial orbits cause RAAN to become meaningless/unstable and, thus, dramatic variations occur in ω and Ω [5]. In this study, this instability is alleviated by rotating the orbital reference plane. This technique is simply to rotate the orbital reference plane by a rotation angle ($\alpha^{\circ }$) from the equatorial plane [14,20]. After the rotation, the satellite position vector $r_{R}$ can be expressed as

(6) $$r_{R}=R_{3}\left(GAST\right)\times R_{1}\left(\alpha \right)\times R_{3}\left(-GAST\right)\times r,$$

where r represents the satellite position vector in an Earth-centered inertial (ECI) coordinate system before the rotation, GAST represents the Greenwich apparent sidereal time at the given epoch, and $R_{1}\left(\cdot \right)$ and $R_{3}\left(\cdot \right)$ are rotation matrices about the *x*-axis and *z*-axis, respectively. By tilting the orbital planes of GEO satellites relative to the Earth, GEO can be considered as the IGSO orbit with inclination (a°).

**Figure 1:**
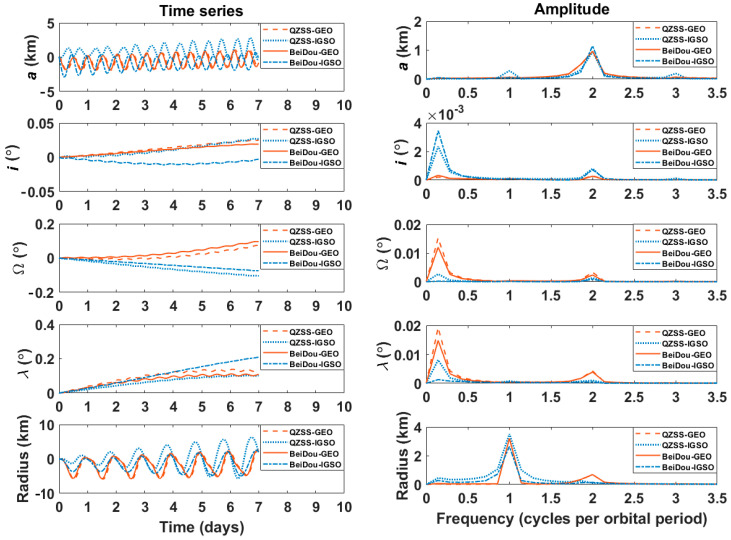
Time variation (**left**) and amplitude spectrum (**right**) of orbital elements: BeiDou geosynchronous (GEO)-C01, BeiDou inclined geosynchronous (IGSO)-C16, QZSS GEO-J01, and QZSS IGSO-J07 satellites.

In order to assess the perturbation effects on IGSO/GEO satellites, Figure 1 presents the variations of their orbital elements. The BeiDou and QZSS satellites were chosen to be representative examples with propagating the high-precision orbital elements for 1 week. This study focuses on the secular and short-periodic variations of orbital elements, since the broadcast ephemerides are typically validated for a short time period, and long-periodic variations can be absorbed into secular and short-periodic variations.

The left panel in Figure 1 shows the deviations of two-body orbital elements (a,i,Ω,λ) and orbital radius (r) from the high-precision model. The GEO orbit was artificially inclined/rotated with $\alpha =5^{\circ }$ through Equation (6). The deviations of each satellite showed a distinctive combination of linear and periodic variations; the periodic variations were more apparent in the semimajor axis (a) and orbital radius (r), whereas the linear variations were more noticeable in the mean longitude (λ). The right panel in Figure 1 shows the amplitude spectrum of periodic variations. The linear variations of the orbital elements were deliberately detrended to assess their periodic characteristics clearly before spectrum analysis. It can be seen that the dominating spectral lines for short-periodic variation in the orbital radius were within the frequencies of 1ϕ and 2ϕ, where ϕ denotes the frequency of satellite motion. The dominating spectral lines for short-periodic variation of other elements were centered in the frequencies of 2ϕ and 3ϕ. The short-periodic variations of orbital elements were mainly due to nonspherical gravitational force and solar radiation pressure. Their effects were superimposed with each other. Specifically, the short-term variations of (a,Ω,λ,r) were caused by the zonal terms of the Earth’s gravitational force and luni-solar attractions, and the short-term variations of (i) were caused by solar radiation pressure. The detailed analysis on the periodic motion of orbital elements can be found in the literature [19,21,22]. These analyses allowed us to design a user algorithm for reconstructing the broadcast ephemeris by taking into account the dominant frequency and characteristics of orbital elements for IGSO/GEO satellites.

### 2.2. Design of Broadcast Ephemeris Parameters

The six Keplerian elements are not enough to satisfy the accuracy requirement of navigation satellites and, thus, additional correction terms are required to describe the linear and periodic variations of orbital elements in general. The classical GPS LNAV ephemeris uses 16 parameters, including three linear correction terms and six periodic correction terms. For more accurate satellite positioning, the GPS CNAV ephemeris was presented by adding the rate of semimajor axis and the rate of mean motion to GPS LNAV [23].

This study aimed to design broadcast ephemeris parameters suited for IGSO/GEO satellites. Considering the compatibility and adaptability with other GNSS orbits, our proposed ephemeris was designed on the basis of GPS LNAV ephemeris. By containing additional correction terms and nonsingular elements to GPS LNAV ephemeris, the variation of orbital elements could be more accurately described without singularity and without sacrificing URE performance. Table 1 shows the six nonsingular elements and 38 additional correction terms used in this study.

Through these parameters, the user algorithm for reconstructing the broadcast ephemeris could compute the satellite positions in ECEF as follows:

(7) $$a\left(t\right)={\left(\sqrt{a_{0}}\right)}^{2}+\dot{a}\left(t-t_{oe}\right)+\frac{1}{2}\ddot{a}{\left(t-t_{oe}\right)}^{2},$$

(8) $$n\left(t\right)=\sqrt{\frac{\mu }{a_{}^{3}\left(t\right)}}+\Delta n+\dot{n}\left(t-t_{oe}\right)+\frac{1}{2}\ddot{n}{\left(t-t_{oe}\right)}^{2},$$

where $t_{oe}$ is the reference epoch and μ denotes the gravitational parameter of the Earth. The eccentricity (ecc), mean anomaly ($M_{0}$), and argument of perigee ($\omega_{0}$) at the reference epoch ($t_{oe}$) were converted from the nonsingular elements as follows:

(9) $$ecc=\sqrt{e_{x}^{2}+e_{y}^{2}},\omega_{0}={tan}^{-1}(e_{y}/e_{x}),M_{0}=\lambda_{0}-\omega_{0}.$$

Mean anomaly (M), eccentric anomaly (E), and true anomaly (ν) could be computed as

(10) $$M=M_{0}+n\left(t\right)\left(t-t_{oe}\right),$$

(11) E=M+eccsinE,

(12) $$\nu =2{tan}^{-1}\left(\frac{\sqrt{1+ecc}}{\sqrt{1-ecc}}tan\frac{E}{2}\right).$$

The periodic correction terms for the argument of latitude (u), orbital radius (r), inclination (i), and RAAN (Ω) were defined as

(13) $$u\left(t\right)=\hat{u}+\dot{u}\left(t-t_{oe}\right)+\frac{1}{2}\ddot{u}{\left(t-t_{oe}\right)}^{2}+Cus1sin\left(\hat{u}\right)+Cuc1cos\left(\hat{u}\right)+Cus2sin\left(2\hat{u}\right)\;+Cuc2cos\left(2\hat{u}\right)+Cus3sin\left(3\hat{u}\right)+Cuc3cos\left(3\hat{u}\right),$$

(14) $$r\left(t\right)=r_{0}+\dot{r}\left(t-t_{oe}\right)+\frac{1}{2}\ddot{r}{\left(t-t_{oe}\right)}^{2}+Crs1sin\left(\hat{u}\right)+Crc1cos\left(\hat{u}\right)+Crs2sin\left(2\hat{u}\right)\;+Crc2cos\left(2\hat{u}\right)+Crs3sin\left(3\hat{u}\right)+Crc3cos\left(3\hat{u}\right),$$

(15) $$i\left(t\right)=i_{0}+\dot{i}\left(t-t_{oe}\right)+\frac{1}{2}\ddot{i}{\left(t-t_{oe}\right)}^{2}+Cis1sin\left(\hat{u}\right)+Cic1cos\left(\hat{u}\right)+Cis2sin\left(2\hat{u}\right)\;+Cic2cos\left(2\hat{u}\right)+Cis3sin\left(3\hat{u}\right)+Cic3cos\left(3\hat{u}\right),$$

(16) $$\Omega \left(t\right)=\Omega_{0}+\dot{\Omega }\left(t-t_{oe}\right)+\frac{1}{2}\ddot{\Omega }{\left(t-t_{oe}\right)}^{2}+C\Omega s1sin\left(\hat{u}\right)+C\Omega c1cos\left(\hat{u}\right)+C\Omega s2sin\left(2\hat{u}\right)\;+C\Omega c2cos\left(2\hat{u}\right)+C\Omega s3sin\left(3\hat{u}\right)+C\Omega c3cos\left(3\hat{u}\right).$$

The orbital radius ($r_{0}$) and uncorrected argument of latitude ($\hat{u}$) at the reference epoch were respectively obtained as follows:

(17) $$r_{0}=a\left(t\right)\left(1-eccE\right),$$

(18) $$\hat{u}=\omega_{0}+\nu .$$

Finally, the satellite position vector in the ECEF coordinate system was given by

(19) $${\vec{r}}_{ECEF}=R_{3}\left(-\Omega \left(t\right)+\omega_{e}\cdot \left(t-t_{oe}\right)\right)R_{1}\left(-i\left(t\right)\right)\left(\begin{array}{c} r\left(t\right)cosu\left(t\right)\\ r\left(t\right)sinu\left(t\right)\\ 0 \end{array}\right),$$

where $\omega_{e}$ denotes the rotation rate of the Earth. For GEO satellites, the position vector ($r_{R}$) after rotation should be rotated back to the position vector (r) before rotation with rotation angle ($-\alpha^{\circ }$) as follows:

(20) $$r=R_{3}\left(GAST\right)\times R_{1}\left(-\alpha \right)\times R_{3}\left(-GAST\right)\times r_{R}.$$

The above equations formed our user algorithm for generating parameter values for broadcast ephemeris through least-squares curve fit. Algorithm 1 describes the step-by-step process of our user algorithm. The computational complexity of this algorithm should be slightly higher than that of GPS LNAV algorithm.
**Algorithm 1:** Procedures for Least-Square Curve Fit of Broadcast Ephemeris Parameters01**Begin**02**Set** simulation period, fit interval, sample rate, and true position/velocity for SVs.
For GEO, the position vector should be rotated with rotation angle by Equation (6).03**Set** initial guesses of broadcast ephemeris parameters for the least-square curve fit. Keplerian elements are converted into the nonsingular elements by Equation (4).04**Main Simulation (User algorithm)**05**For***i* = *t_0_*: *t_0_* + fit interval06  Nonsingular elements are converted into Keplerian elements by Equation (5).07  The IGSO/GEO position vector at *t*(*i*) is computed through Equations (7)–(19).09 **End for**10 For GEO, the position vectors should be rotated back by Equation (20).11Compute the fit accuracy of broadcast ephemerides through comparison with the true position at time interval.

The ephemeris parameters involved in the user algorithm were classified as compulsory and optional ones, as shown in Table 2. The 16 compulsory parameters were similar to GPS LNAV ephemeris parameters. The 28 optional parameters were selected to precisely represent the dominant linear and periodic variations of orbital elements. The parameter values of broadcast ephemeris were computed using a least-squares curve fit as

(21) $$Y=\overset{-}{Y}\left(X,t,t_{oe}\right)+\varepsilon ,$$

where ε represents the residual. Y denotes the vector of observation with time t, and X denotes the proposed 44 broadcast ephemeris parameters at $t_{oe}$. The partial derivative matrix H was as follows:

(22) $$H=\frac{\partial Y}{\partial X}=\left(\frac{\partial Y}{\partial \sqrt{a_{0}}}\cdots \frac{\partial Y}{\partial Cis3}\right).$$

Compared to GPS LNAV/CNAV ephemeris parameters, only three Keplerian elements (ecc,ω,M) were replaced with nonsingular elements in our proposed broadcast ephemeris parameters. With respect to the nonsingular elements, the partial derivatives of the satellite position vector in ECEF coordinates were derived as follows:

(23) $$\frac{\partial r_{ECEF}}{\partial e_{x}}=\left(\frac{\partial r_{ECEF}}{\partial r}\right)\left(\frac{\partial r}{\partial e_{x}}\right)=R_{3}\left(\omega_{e}\cdot \left(t-t_{oe}\right)\right)\cdot \left(P\cdot r+Q\cdot \dot{r}\right)\;\frac{\partial r_{ECEF}}{\partial e_{y}}=\left(\frac{\partial r_{ECEF}}{\partial r}\right)\left(\frac{\partial r}{\partial e_{y}}\right)=R_{3}\left(\omega_{e}\cdot \left(t-t_{oe}\right)\right)\cdot \left(R\cdot r+S\cdot \dot{r}\right)\;\frac{\partial r_{ECEF}}{\partial \lambda }=\left(\frac{\partial r_{ECEF}}{\partial r}\right)\left(\frac{\partial r}{\partial \lambda }\right)=R_{3}\left(\omega_{e}\cdot \left(t-t_{oe}\right)\right)\cdot \frac{\dot{r}}{n},$$

where the pair ($r,\dot{r}$) denotes the satellite position and velocity in ECI coordinates, respectively. The coefficients (*P*, *Q*, *R*, and *S*) were given as follows:

(24) $$P=\frac{a}{p}\left[-\left(cosu+e_{x}\right)-\frac{r}{p}\left(sinu+e_{y}\right)\left(e_{x}sinu-e_{y}cosu\right)\right]\;Q=\frac{ar}{\sqrt{\mu p}}\left[sinu+\frac{ae_{y}\sqrt{1-ecc^{2}}}{r\left(1+\sqrt{1-ecc^{2}}\right)}+\frac{r}{p}\left(sinu+e_{y}\right)\right]\;R=\frac{a}{p}\left[\left(sinu+e_{y}\right)-\frac{r}{p}\left(cosu+e_{x}\right)\left(e_{x}sinu-e_{y}cosu\right)\right]\;S=-\frac{ar}{\sqrt{\mu p}}\left[cosu+\frac{ae_{x}\sqrt{1-ecc^{2}}}{r\left(1+\sqrt{1-ecc^{2}}\right)}+\frac{r}{p}\left(cosu+e_{x}\right)\right],$$

where $p=a\left(1-ecc^{2}\right)$. The partial derivatives of other parameters could be derived from the user algorithm [15,24,25]. Initial guesses for design variables are prerequisite in the iterative curve fit. In this study, initial guesses were determined such that those for nonsingular elements were osculating orbital elements and those for the other parameters were zero. Since computational memory is limited and the amplitudes of some parameters are negligibly small, the optimal parameter set of broadcast ephemeris should be carefully determined. This study employed the experiment process in the literature [14,15] to consider all possible parameter selections. The accuracy of broadcast ephemerides was determined by the selected parameters. On the basis of 16 compulsory parameters, we selected one to four parameters from the optional group and then compared their fit accuracy. The terms in Equations (7)–(24) could be accepted or rejected according to the selected parameters. The optional group had 28 parameters consisting of 10 linear terms and nine pairs of harmonic terms. The additional parameters were selected with the condition that sine and cosine harmonic terms should be selected as a pair. Table 3 summarizes the number of possible sets of parameters. Among all possible parameter sets, the parameter set that provided the minimum fit error was determined as the optimal broadcast ephemeris parameters. The fit performance of ephemerides was obtained in an Earth-centered inertial (ECI) coordinate system because the performance of curve-fitting could be affected by transformation into the ECEF frame [14]. The associated results and analysis for possible parameter sets are discussed in Section 3.

## 3. Results and Analysis

This section is dedicated to analyzing the fit performance and selecting the set of optimal parameters in terms of accuracy and the number of parameters. The URE (user range error), which is useful to measure the fit accuracy, is defined as a weighted average of the root-mean-square (RMS) errors for the three RTN components.

(25) $$\mathrm{URE}≜\sqrt{w_{A}^{2}R^{2}+w_{B,C}^{2}\left(A^{2}+C^{2}\right)},$$

where R≜RMS(ΔR), A≜RMS(ΔT), and C≜RMS(ΔN) are RMS errors of the radial, along-track, and cross-track components, respectively [26]. The pair weighted factors ($w_{A},w_{B,C}$) depend on the orbital altitude. Referring to Montenbruck et al. [27], which addressed the proper values of weighted factors for IGSO/GEO satellites of BeiDou, we set ($w_{A},w_{B,C}$) = (0.99, 1/126).

### 3.1. Parameter Selection and Fit Accuracy

The accuracy of broadcast ephemeris is subject to orbit determination errors, propagation errors, and fit errors [28]. Only the fit error, which was evaluated by URE, was addressed in this study. The accuracy of least-square curve fit was analyzed in terms of fit interval and the number of parameters. Chosen for accuracy analysis were four GEO (PRN: C01, C02, C04, C05) and six IGSO (PRN: C06, C07, C09, C10, C13, C16) satellites of BeiDou. The precise ephemerides determined from the pseudorange and carrier phase observables were used as true/reference values for statistical analysis. The simulation period was 24 h, starting at 00:00:00 GPST on the first day of the year 2019 (31 December 2018, 23:59:42 UTC). The least-square curve fit was carried out with 2 and 3 h fit intervals with a uniform sample rate of 300 s. Thus, for example, 12 fitting arcs were generated per day with a 2 h fitting interval, and one arc contained 24 data points.

Table 4 and Table 5 list some representative results of adding one to four parameters. Table 4 shows the RMS of RTN component errors and the URE for PRN: C07 (or BeiDou-IGSO2). The best results among the same number of parameter sets are shown in bold. Compared to the set of 16 compulsory parameters, the second-order rates of radius ($\ddot{r}$) and semimajor axis ($\ddot{a}$) influenced the fit accuracy in the radial and along-track but not cross-track directions. Similarly, in the case of adding two optional parameters, the first and third orders of harmonic corrections to the argument of latitude (*Cus*, *Cuc*) and radius (*Crs*, *Crc*) affected the fit accuracy in the radial and along-track but not cross-track directions. However, the harmonic correction terms for RAAN had a significant effect on the fit accuracy of cross-track direction in the sense that RMS errors of the cross-track direction with (CΩS3,CΩC3), (CΩS2,CΩC2), and (CΩS1,CΩC1) varied. The minimum fit errors in terms of URE were obtained by adding ($\dot{r},\ddot{r}$). In the case of adding three and four parameters, it can be seen that the parameter sets containing the first-order and/or second-order rates of radius and semimajor axis had higher performance than the others. The optimal additions for 19- and 20- parameter sets were ($\dot{a},\dot{r},\ddot{r}$) and ($\dot{r},\ddot{r},$ *Crs3*, *Crc3*), respectively. In all the cases, the terms related to inclination and mean motion had relatively small effects on the fit accuracy.

Table 5 lists the RMS of RTN components and the URE for PRN: C02 (or BeiDou-GEO6). Compared to the set of 16 compulsory parameters, the first-rate ($\dot{a}$) and second-rate ($\ddot{a}$) terms of the semimajor axis affected the fit accuracy in the radial and along-track but not cross-track directions. In the case of adding two optional parameters, unlike the fit results of IGSO in Table 4, the first-rate and second-rate terms of the semimajor axis and radius predominantly influenced the fit accuracy on radial and along-track directions. The optimal parameters turned out to be (*Crs3*, *Crc3*). In the cases of adding three and four optional parameters, the minimum fit errors were achieved by adding (*Crs3*, *Crc3*,$\ddot{r}$) and ($\dot{a},\dot{r},$ *Crs3*, *Crc3*), respectively. Similar to the fit results of IGSO, the fit errors in the cross-track direction were still dependent on harmonic correction terms of RAAN, such as (CΩS1,CΩC1), (CΩS1,CΩC1,$\dot{r}$), and (Crs3, Crc3,CΩS3,CΩC3). The correction terms for inclination and mean motions still had little impact on minimizing the URE. Table 4 and Table 5 reveal the common additional parameters sets for both IGSO and GEO to be ($\ddot{a}$), (CΩS1,CΩC1), ($\dot{a},\dot{r},\ddot{a}$), and ($\dot{r},\ddot{r},$ *Crs3*, *Crc3*). From the perspective of operating GNSS, it should provide simplicity, uniformity, and compatibility of the user algorithm to use a sole type of broadcast ephemeris parameters. In this sense, we selected the common additional parameters which were not suited for either IGSO or GEO, but which were suited for both IGSO and GEO as the optimal set of parameters. The numerical experiments and analyses showed that these parameter sets were effective and practical to minimize fit errors.

### 3.2. Analysis of Fit Accuracy

**Figure 2:**
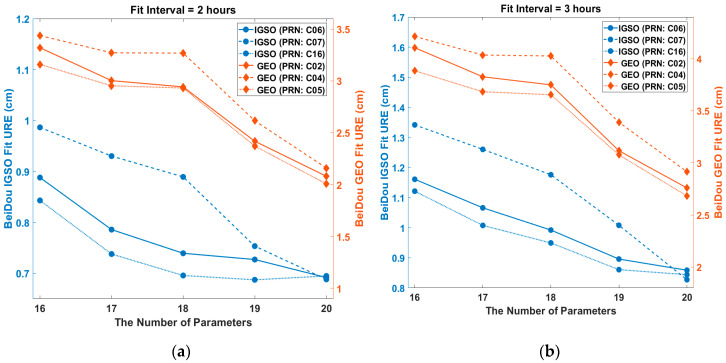
Fit URE for BeiDou IGSOs (PRN: C06, C07, and C16) and GEOs (PRN: C02, C04, and C05) vs. the number of parameters: (**a**) 2 h fit interval; (**b**) 3 h fit interval.

Figure 2 shows the relationship between URE and the number of parameters for different types of orbit. It can be seen that UREs decreased clearly as the number of parameters increased. The URE of the 19-parameter set was significanlty reduced compared to that of the 16-parameter set. In particular, for GEOs and IGSO (RPN: C07), their URE reduction from 18 to 19 parameters was steep. The improvement in the radial direction mainly caused URE reductions. This shows that the radial movements could be more accurately described by adding a few appropriate parameters in the ephemeris. Since the URE reduction slope from 19 to 20 parameters was relatively small, it was reasonable to choose a 19-parameter set for the optimal broadcast ephemeris.

**Figure 3:**
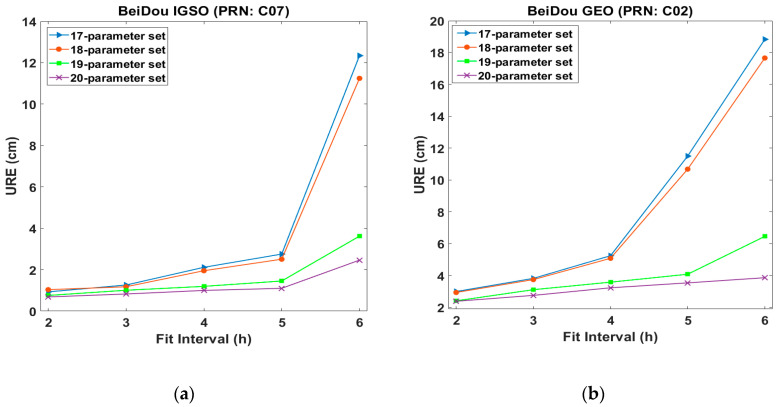
UREs vs. fit intervals: (**a**) BeiDou IGSO (PRN: C07); (**b**) BeiDou GEO (PRN C02).

Figure 3 illustrates the relationship between the URE and the fit intervals. For both IGSO and GEO satellites, the increase in URE between the 5 and 6 h fit intervals was significant. A smaller number of parameters led to a steeper slope of the URE as the fit interval increased. The UREs for all parameter sets were less than 1.03 cm for IGSO and 3.00 cm for GEO with a 2 h fit interval. With a 6 h fit interval, the URE for the 17-parameter set skyrocketed from 2.7 to 12.3 cm for IGSO and from 11.5 to 18.8 cm for GEO. For the 20-parameter set, the URE was maintained at less than 2.4 cm for IGSO and less than 3.8 cm for GEO in all fit intervals.

**Figure 4:**
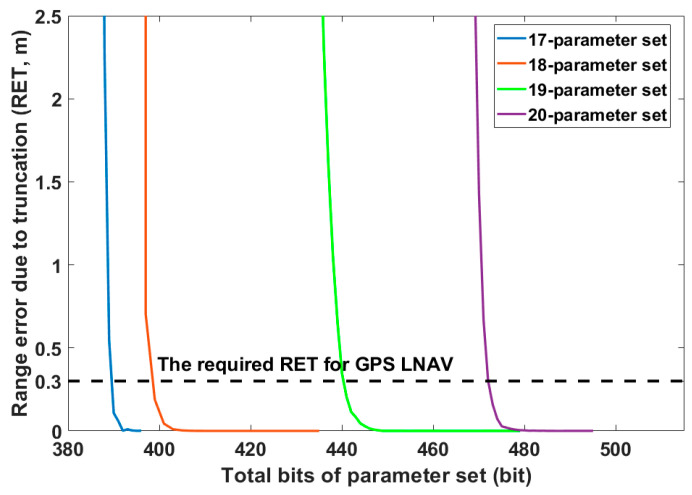
Total bits vs. RET for different ephemeris parameter sets.

The bit allocation to the ephemeris parameter and analysis of the range error due to truncation (RET) are worth being investigated in the practical design of navigation messages. Hence, the relationship between the total bits of the proposed ephemerides and range error of URE due to truncation is presented. The bit length could be obtained using the arithmetic relationship between scale factor and effective range. Through the variations of the scale factor value, the sensitivity analysis of RET was performed to determine the least significant bits (LSBs) for the proposed ephemerides [2]. Figure 4 shows the total bits for our proposed ephemerides according to RET. It indicates that their total bits were higher than the classical BeiDou ephemeris with 371 bits [9]. As the total number of bits of the parameter sets decreased and reached a critical point, the RET increased exponentially. On the other hand, as the total number of bits of parameter sets increased beyond a critical point, the RET converged gradually to a particular value. Between the 18- and 19-parameter sets, the increase in the total number of bits for obtaining the equivalent RET was more noticeable. Since the RET of the GPS LNAV is required to be less than 0.3 m, the LSBs of the proposed 17-, 18-, 19-, and 20-parameter sets were set such that RET was less than 0.3 m [2]. Their values were 390, 399, 441 and 472 bits, respectively. Note that the total number of bits of the 18-parameter set was lower than for GPS CNAV, i.e., 421 bits for 18 ephemeris parameters [29].

### 3.3. Comparison with Other Ephemerides

Our proposed broadcast ephemeris sets were compared with four different ones, including the conventional/well-known BeiDou ephemeris [9], GPS CNAV [23], and those proposed by Fu et al. [14]. Table 6 describes these ephemerides models in detail. A least-square curve fit was conducted with a 2 h fit interval and 300 s of sample rate for the IGSO/GEO satellites of BeiDou.

Table 7 summarizes the fit UREs for each satellite and their RMS. Compared to the classical BeiDou ephemeris, our proposed parameter sets 1–4 composed of 17–20 parameters showed better URE performance for all satellites. Their RMS values of UREs were 6.27%, 7.58%, 23.8%, and 34.5% smaller than those of the classical BeiDou ephemeris; the UREs could be significantly improved by adding a few ephemeris parameters. Even when compared with other models, our proposed parameter sets showed competitive fit performance. GPS CNAV and the proposed parameter set 2 had the same number of ephemeris parameters, but the difference in the RMS value of UREs was 0.532 cm. The proposed parameter set 2 showed 21% better fit accuracy than GPS CNAV. Fu et al. [14] 2 and the proposed parameter set 1 had an identical (17) number of parameters, but their composition of parameters was different, as was the contribution of each parameter in describing the orbital perturbation. The maximum and minimum UREs for Fu et al. [14] 2 were, respectively, 3.934 and 0.599 cm for BeiDou satellites, while the maximum and minimum UREs for the proposed parameter set 1 were 3.271 and 0.572 cm, respectively.

### 3.4. Broacast Ephemeris Fit for QZSS

Our proposed ephemeris parameter sets 1–4 were also tested with three QZSS IGSOs (PRN J01-03) and one QZSS GEO (PRN: J07). QZSS observation data were used as the true/reference values for comparison. The period was from 31 December 2018, 23:59:42 UTC to 1 January 2019, 23:59:42 UTC.

**Figure 5:**
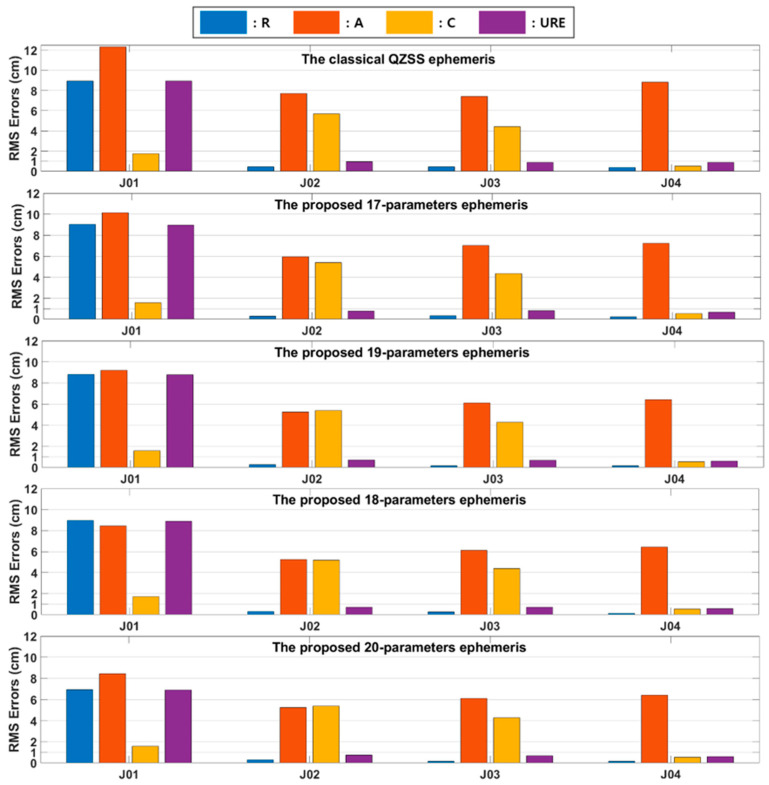
RMS errors in RTN components and UREs with different parameter sets for QZSS.

The least-square curve fit was performed with a 2 h fit interval and 300 s of sample rate. QZSS GEO satellite (PRN: J07) was positioned nearby 127° east (E), while BeiDou GEO satellites (PRN: C01-C05) were distributed nearby 140°, 80°, 110.5°, 160°, and 58.75° E [30,31]. Figure 5 shows the fit errors of RTN components and UREs for our proposed ephemeris 1–4 composed of 17-20 parameters.

For IGSO satellites, our parameter sets 1–4 ensured that RMS errors of R/T/N components were less than 8.9/10.1/1.72 cm for J01, 3.0/5.9/5.3 cm for J02, and 3.0/7.0/4.3 cm for J03. For GEO satellites, our parameter sets 1–4 achieved RMS errors of R/T/N components less than 0.2/7.2/0.54 cm. Unlike BeiDou, as the number of ephemeris parameters increased from 17 to 20, decreases in fit errors were not clearly observed. The URE of conventional QZSS ephemeris (GPS LNAV) is 8.9 cm [10]. Our proposed ephemeris parameter sets 1–4 achieved fit UREs of 8.9, 8.8, 8.7 and 6.9 cm, respectively.

## 4. Conclusions

We presented an optimal set of broadcast ephemeris parameters for IGSO/GEO satellites (in terms of URE). Their orbital characteristics were carefully adapted to establish a new set of ephemeris parameters efficiently accommodating the impact of dominant perturbations in high Earth orbit. Those sets were generated by adding 1–4 parameters to GPS LNAV ephemeris so that the core of the GPS LNAV user algorithm remained unchanged. All possible ephemeris parameter sets were tested with BeiDou satellites, showing that the minimum URE could be achieved by adding ($\ddot{r}$), ($\dot{r},\ddot{r}$), ($\dot{a},\dot{r},\ddot{r}$), and ($\dot{r},\ddot{r},$ Crs3, Crc3) for IGSO satellites, and ($\dot{a}$), (Crs3, Crc3), (Crs3, Crc3, $\ddot{r}$), and ($\dot{a},\dot{r},$ Crs3, Crc3) for GEO satellites. Adding ($\dot{r},\ddot{r},$ Crs3, Crc3) for IGSO and ($\dot{a},\dot{r},$ *Crs*3, *Crc*3) for GEO led to UREs of 0.688 and 1.97 cm for the 2 h fit interval, respectively. We also investigated how the number of parameters and fit intervals affected the fit accuracy. Some noticeable improvement in URE was observed when the number of parameters was changed from 18 to 19 and the fit interval was reduced from a 6 to 5 h interval. Additionally, RET was evaluated with reference to the total number of bits of the proposed ephemeris parameter sets. The total number of bits of the proposed 17- and 18-parameter sets was lower than that of GPS CNAV, and only a few more bits were needed for the classical BeiDou ephemeris to improve URE accuracy. However, the 19- and 20-parameter sets, with significantly improved accuracy, required an additional 70 and 101 bits, respectively, in the navigation message due to parameter additions.

We presented the common parameter sets between IGSO and GEO, considering compatibility and uniformity. Their fit performances were compared with four different well-known ephemeris models. Statistical results showed that our proposed parameter sets achieved a minimum RMS of UREs of 1.928 cm for the 17-parameter set and 1.346 cm for the 20-parameter set, representing roughly 6.27% and 34.5% improvements. The proposed ephemerides were also validated with four QZSS satellites for generality. Although empirically analyzed, our proposed ephemerides were effective in terms of fit capability of the IGSO and GEO satellites and the avoidance of singularities caused by small eccentricity/inclination. It is natural and straightforward to extend its applicability to other GNSS composed of IGSO/GEO satellites.

## Figures and Tables

**Table 1:** Parameters involved in the proposed algorithm. RAAN, right ascension of the ascending node.

Parameters	Description
$t_{oe}$	Ephemeris reference epoch
($a_{0},\dot{a},\ddot{a}$)	Semimajor axis at reference epoch and its first-order and second-order rates
($e_{x},e_{y}$)	Two-dimensional components of eccentricity
($\Omega_{0},\dot{\Omega },\ddot{\Omega }$)	RAAN at reference epoch, and its first-order and second-order rates
($i_{0},\dot{i},\ddot{i}$)	Inclination at reference time, and its first-order and second-order rates
($\dot{u},\ddot{u}$)	The first-order and second-order rates of AOL
($\dot{r},\ddot{r}$)	The first-order and second-order rates of orbital radius
Δn	Mean motion difference from the calculated value at $t_{oe}$
($\dot{n},\ddot{n}$)	The first-order and second-order rates of mean motion
λ	Mean argument of latitude
(Crc1,Crs1,Crc2,Crs2,Crc3,Crs3)	Amplitude of the first, second, and third cosine and sine harmonic correction terms to the orbital radius
(Cuc1,Cus1,Cuc2,Cus2,Cuc3,Cus3)	Amplitude of the first, second, and third cosine and sine harmonic correction terms to the AOL
(CΩc1,CΩs1,CΩc2,CΩs2,CΩc3,CΩs3)	Amplitude of the first, second, and third cosine and sine harmonic correction terms to the RAAN
(Cic1,Cis1,Cic2,Cis2,Cic3,Cis3)	Amplitude of the first, second, and third cosine and sine harmonic correction terms to the inclination

**Table 2:** Compulsory and optional parameters for broadcast ephemeris.

Attribute	Compulsory Parameters	Optional Parameter
Reference epoch	*t_oe_*	
Orbital elements	($\sqrt{a},e_{x},e_{y},i,\Omega ,\lambda$)	
Secular-periodic correction	($\Delta n,\dot{i},\dot{\Omega }$)	($\dot{a},\dot{n},\dot{r},\dot{u},\ddot{a},\ddot{n},\ddot{r},\ddot{u},\ddot{\Omega },\ddot{i}$)
Short-periodic correction	(*Cuc2*, *Cus2*, *Crc2*, *Crs2*, *Cic2*, *Cis2*)	(*Cuc3*, *Cus3*, *Cuc1*, *Cus1*, *Cic3*, *Cis3*, *Cic1*, *Cis1*, *Crc3*, *Crs3*, *Crs1*, *Crs1*,CΩc3,CΩS3,CΩc2,CΩS2,CΩc1,CΩS1)

**Table 3:** The number of possible parameter sets according to the number of additional parameters.

Case	Descriptions	Possible Parameter Sets
One additional parameter	Linear terms	_10_C_1_ = 10
Two additional parameters	Two linear terms One pair of harmonic terms	_10_C_2_ + _9_C_1_ = 54
Three additional parameters	Three linear terms One pair of harmonic terms plus one linear term	_10_$C_{3}+{\;}_{10}C_{1}\times$ 9 = 210
Four additional parameters	Four linear terms; two pairs of harmonic terms One pair of harmonic terms plus two linear terms	_10_$C_{4}+{\;}_{9}C_{2}+{\;}_{10}C_{2}\times$ 9 = 651

**Table 4:** Fit user range error (URE) and root-mean-square (RMS) fit errors in radial, along-track, and cross-track directions with 2 and 3 h fit intervals for IGSO satellite (BeiDou PRN: C07). The parts in bold represent the best fit URE for the same number of parameters.

Number of Parameters	Additional Parameter	2 h Fit Interval (cm)				3 h Fit Interval (cm)			
		R	A	C	URE	R	A	C	URE
16	-	0.794	6.161	2.603	0.987	1.154	7.304	3.014	1.342
17	($\ddot{r}$)	**0.733**	**5.012**	**2.603**	**0.883**	**1.026**	**5.895**	**3.014**	**1.175**
	($\ddot{a}$)	0.790	5.013	2.603	0.930	1.125	5.892	3.014	1.260
18	($\dot{r},\ddot{r}$)	**0.704**	**4.387**	**2.603**	**0.832**	**0.983**	**5.308**	**3.014**	**1.115**
	(*Cus3*, *Cuc3*)	0.753	4.401	2.603	0.874	1.060	5.338	3.014	1.183
	(CΩS2,CΩC2)	0.753	4.415	2.732	0.877	1.060	5.370	3.210	1.188
	(CΩS3,CΩC3)	0.752	4.402	2.432	0.869	1.059	5.339	2.773	1.177
	(*Crs1*, *Crc1*)	0.765	4.389	2.603	0.883	1.069	5.309	3.014	1.190
	(CΩS1,CΩC1)	0.777	4.389	2.431	0.889	1.059	5.311	2.770	1.176
19	(*Crs*1, *Crc*1,$\dot{a}$)	0.579	4.388	2.603	0.732	0.818	5.308	3.014	0.975
	($\dot{a},\dot{r},\ddot{r}$)	**0.565**	**4.394**	**2.603**	**0.721**	**0.809**	**5.317**	**3.014**	**0.969**
	($\dot{a},\dot{r},\ddot{a}$)	0.607	4.392	2.603	0.753	0.857	5.315	3.014	1.008
	(*Cus*1, *Cuc*1,$\dot{u}$)	0.633	4.393	2.603	0.774	0.841	5.319	3.014	0.995
	(*Cus*1, *Cuc*1,$\dot{a}$)	0.626	4.396	2.603	0.769	0.861	5.321	3.014	1.012
	(*Cus*1, *Cuc*1,$\dot{r}$)	0.594	4.387	2.603	0.743	0.817	5.308	3.014	0.975
20	($\dot{r},\ddot{r},$ *Crs*3, *Crc*3)	**0.522**	**4.387**	**2.603**	**0.688**	**0.630**	**5.308**	**3.014**	**0.827**
	($\dot{r},\ddot{r},$ *Cus*1, *Cuc*1)	0.580	4.387	2.603	0.732	0.822	5.308	3.014	0.979
	($\ddot{a},\ddot{r},$ *Crs*1, *Crc*1)	0.598	4.384	2.603	0.746	0.825	5.302	3.014	0.981
	($\dot{a},\ddot{r},$ *Crs*1, *Crc*1)	0.572	4.386	2.603	0.726	0.826	5.295	3.014	0.981
	($\dot{r},\ddot{r},$ *Crs*1, *Crc*1)	0.576	4.387	2.603	0.729	0.792	5.308	3.014	0.954
	($\dot{r},\ddot{a},$ *Crs*3, *Crc*3)	0.615	4.370	2.603	0.759	0.768	5.259	3.014	0.932

**Table 5:** Fit URE and RMS fit errors in radial, along-track, and cross-track directions with 2 and 3 h fit intervals for GEO satellite (BeiDou PRN: C02). The parts in bold represent the best fit URE for the same number of parameters.

Number of Parameters	Additional Parameter	2 h Fit Interval (cm)				3 h Fit Interval (cm)			
		R	A	C	URE	R	A	C	URE
16	-	2.847	19.654	1.236	3.320	3.657	21.676	1.603	4.106
17	($\dot{a}$)	**2.595**	**16.580**	**1.235**	**2.965**	**3.396**	**19.657**	**1.598**	**3.793**
	($\ddot{a}$)	2.669	15.964	1.235	3.002	3.453	19.239	1.598	3.827
18	(*Crs3*, *Crc3*)	**1.808**	**13.569**	**1.235**	**2.163**	**2.709**	**17.136**	**1.598**	**3.089**
	($\dot{r},\ddot{a}$)	2.130	13.500	1.235	2.430	2.999	17.010	1.598	3.337
	($\dot{a},\dot{r}$)	2.230	16.539	1.235	2.656	2.924	19.587	1.598	3.383
	($\ddot{a},\ddot{r}$)	2.243	15.934	1.235	2.638	2.811	19.148	1.598	3.267
	($\dot{u},\ddot{r}$)	2.321	13.514	1.239	2.596	2.931	16.966	1.602	3.275
	(CΩS1,CΩC1)	2.708	13.567	1.146	2.942	3.457	17.133	1.485	3.749
19	(*Crs3*, *Crc3*,$\ddot{r}$)	**1.706**	**13.569**	**1.235**	**2.080**	**2.326**	**17.134**	**1.598**	**2.766**
	(*Crs3*, *Crc3*,$\ddot{a}$)	1.716	13.573	1.235	2.088	2.334	17.138	1.598	2.773
	(*Crs3*, *Crc3*,$\dot{r}$)	1.719	13.569	1.235	2.091	2.326	17.134	1.598	2.766
	(*Crs3*, *Crc3*,$\dot{a}$)	1.731	13.574	1.235	2.100	2.347	17.139	1.598	2.784
	($\dot{a},\dot{r},\ddot{a}$)	2.117	13.502	1.235	2.419	2.747	17.029	1.598	3.117
	(CΩS1, CΩC1,$\dot{r}$)	2.138	13.564	1.147	2.440	2.753	17.133	1.481	3.126
20	($\dot{a},\ddot{r},$ *Crs*3, *Crc*3)	1.757	13.372	1.235	2.111	3.821	16.492	1.598	4.061
	($\dot{r},\dot{r},$ *Crs*3, *Crc*3)	**1.631**	**12.609**	**1.234**	**1.970**	**2.053**	**15.111**	**1.598**	**2.442**
	($\dot{r},\ddot{r},$ *Crs*3, *Crc*3)	1.707	13.569	1.235	2.081	2.318	17.134	1.598	2.760
	(*Cus*3, *Cuc*3, *Crs*3, *Crc*3)	1.765	13.544	1.235	2.126	2.429	17.075	1.598	2.849
	($\ddot{a},\ddot{r},$ *Crs*3, *Crc*3)	1.702	13.436	1.235	2.070	2.308	16.908	1.598	2.740
	(*Crs*3, *Crc*3,CΩS3,CΩC3)	1.786	13.545	1.227	2.144	2.406	17.080	1.641	2.830

**Table 6:** Main differences in parameters and characteristics of competitive ephemeris models. GPS, global positioning system.

Ephemeris (No. of Parameters)	Reference Epoch	Orbital Elements	Secular Correction	Periodic Correction
BeiDou ephemeris	$\left(t_{0}\right)$	Quasi-Kepler elements	$\left(\Delta n,\dot{\Omega },\dot{i}\right)$	(*Cus2*, *Cuc2*, *Crs2*)
(16)		$\left(\sqrt{a},ecc,i_{0},\Omega_{0},\omega_{0},M_{0}\right)$		(*Crc2*, *Cis2*, *Cic2*)
GPS CNAV	$\left(t_{0}\right)$	Quasi-Kepler elements	$\left(\dot{a},\dot{i}\right)$ $\left(\Delta n,\Delta \dot{n},\Delta \dot{\Omega }\right)$	(*Cus2*, *Cuc2*, *Crs2*)
(18)		$\left(\Delta a_{0},ecc,i_{0},\Omega_{0},\omega_{0},M_{0}\right)$		(*Crc2*, *Cis2*, *Cic2*)
Fu et al. [14] 1	$\left(t_{0}\right)$	Quasi-Kepler elements	$\left(\dot{\Omega },\dot{u},\dot{i}\right)$	(CΩS3,CΩC3, *Crs2*)
(16)		$\left(\sqrt{a_{0}},ecc_{0},i_{0},\Omega_{0},\omega_{0},M_{0}\right)$		(*Crc2*, *Cus2*, *Cuc2*)
Fu et al. [14] 2	$\left(t_{0}\right)$	Quasi-Kepler elements	$\left(\dot{\Omega },\dot{u},\dot{r},\dot{i}\right)$	(CΩS1,CΩC1, *Crs2*)
(17)		$\left(\sqrt{a_{0}},ecc_{0},i_{0},\Omega_{0},\omega_{0},M_{0}\right)$		(*Crc2*, *Cus2*, *Cuc2*)
Proposed set 1	$\left(t_{0}\right)$	Nonsingular elements	$\left(\ddot{a}\right)$ $\left(\Delta n,\dot{\Omega },\dot{i}\right)$	(*Cus2*, *Cuc2*, *Crs2*)
(17)		$\left(\sqrt{a_{0}},e_{x},e_{y},i_{0},\Omega_{0},\lambda_{0}\right)$		(*Crc2*, *Cis2*, *Cic2*)
Proposed set 2	$\left(t_{0}\right)$	Nonsingular elements	$\left(\Delta n,\dot{\Omega },\dot{i}\right)$	(CΩS1,CΩC1, *Cus2*, *Cuc2*)
(18)		$\left(\sqrt{a_{0}},e_{x},e_{y},i_{0},\Omega_{0},\lambda_{0}\right)$		(*Crs2*, *Crc2*, *Cis2*, *Cic2*)
Proposed set 3	$\left(t_{0}\right)$	Nonsingular elements	$\left(\dot{a},\ddot{a},\dot{r}\right)$ $\left(\Delta n,\dot{\Omega },\dot{i}\right)$	(*Cus2*, *Cuc2*, *Crs2*)
(19)		$\left(\sqrt{a_{0}},e_{x},e_{y},i_{0},\Omega_{0},\lambda_{0}\right)$		(*Crc2*, *Cis2*, *Cic2*)
Proposed set 4	$\left(t_{0}\right)$	Nonsingular elements	$\left(\dot{r},\ddot{r}\right)$ $\left(\Delta n,\dot{\Omega },\dot{i}\right)$	(*Cus2*, *Cuc2*, *Crs3*, *Crc3*)
(20)		$\left(\sqrt{a_{0}},e_{x},e_{y},i_{0},\Omega_{0},\lambda_{0}\right)$		(*Crs2*, *Crc2*, *Cis2*, *Cic2*)

**Table 7:** UREs and their RMS values for difference ephemeris models.

Ephemeris Model	BeiDou GEO UREs (cm)				BeiDou IGSO UREs (cm)						RMS
	C01	C02	C04	C05	C06	C07	C09	C10	C13	C16	(cm)
BeiDou ephemeris	2.477	3.296	3.341	3.136	0.833	0.943	0.832	0.630	0.704	0.785	2.057
GPS CNAV	2.373	3.138	3.991	3.835	1.204	1.683	1.127	1.933	1.682	1.029	2.433
Fu et al. [14] 1	2.328	3.100	3.287	3.068	0.884	0.922	0.751	0.637	0.679	0.828	2.009
Fu et al. [14] 2	1.927	2.929	3.001	3.934	0.830	0.830	0.823	0.599	0.748	0.794	1.975
Proposed set 1	2.318	3.002	3.271	2.953	0.786	0.930	0.768	0.572	0.649	0.738	1.928
Proposed set 2	2.300	2.942	3.267	2.931	0.739	0.889	0.706	0.531	0.567	0.696	1.901
Proposed set 3	1.881	2.419	2.616	2.372	0.727	0.753	0.710	0.499	0.586	0.687	1.566
Proposed set 4	1.594	2.080	2.160	2.007	0.692	0.688	0.709	0.522	0.586	0.695	1.346

## References

[1] Montenbruck O., Hauschild A., Steigenberger P., Hugentobler U., Teunissen P., Nakamura S. (2012). Initial assessment of the COMPASS/BeiDou-2 regional navigation satellite system. GPS Solut..

[2] Wang L.X., Huang Z.G., Zhao Y. (2014). Navigation message designing with high accuracy for NAV. Chin. J. Aeronaut..

[3] Lv Y., Geng T., Zhao Q., Xie X., Zhou R. (2020). Initial assessment of BDS-3 preliminary system signal-in-space range error. GPS Solut..

[4] Heng L., Gao G., Walter T., Enge P. Statistical characterization of GLONASS broadcast ephemeris errors. Proceedings of the 24th International Technical Meeting of the Satellite Division of the Institute of Navigation (ION GNSS 2011).

[5] Roy A.E. (1988). Orbital Motion.

[6] Xu G., Xu J. (2013). On the singularity problem in orbital mechanics. Month. Not. Royal Astronom. Soc..

[7] Cohen C.J., Hubbard E.C. (1962). A Nonsingular Set of Orbital Elements. Astronom. J..

[8] European Union 2016, Signal-In-Space Interface Control Document, OS SIS ICD, Issue 1.3, 2016. https://www.gsc-europa.eu/sites/default/files/sites/all/files/Galileo-OS-SIS-ICD.pdf.

[9] BeiDou ICD (2018). BeiDou Navigation Satellite System Signal in Space Interface Control Document Open Service Signal B3I (Version 1.0). http://en.beidou.gov.cn/beidoupolicy.html.

[10] Cabinet Office, Quasi-Zenith Satellite System Services Inc. (2018). Quasi-Zenith Satellite System Interface Specification Satellite Positioning, Navigation and Timing Service (IS-QZSS-PNT-003). https://qzss.go.jp/en/technical/ps-is-qzss/ps-is-qzss.html.

[11] Montenbruck O., Steigenberger P., Riley S. (2015). IRNSS orbit determination and broadcast ephemeris assessment. Inst. Navig. Int. Tech. Meet..

[12] Qin Z., Huang G., Zhang Q., Wang L., Yan X., Kang Y., Wang X., Xie S. (2019). A Method to Determine BeiDou GEO/IGSO Orbital Maneuver Time Periods. Sensors.

[13] Keyvani F., Torabi S.H. (2019). Design and Simulation of Regional Navigation Constellation with Optimized Mean DOP based on Hybrid GEO and IGSO Satellites. Int. J. Aviation Aeronaut. Aerospace.

[14] Fu X., Wu M. (2011). Optimal design of broadcast ephemeris parameters for a navigation satellite system. GPS Solut..

[15] Xie X., Geng T., Zhao Q., Liu X., Zhang Q., Liu J. (2018). Design and validation of broadcast ephemeris for low Earth orbit satellites. GPS Solut..

[16] Du L., Zhang Z., Zhang J., Liu L., Guo R., He F. (2014). An 18-element GEO broadcast ephemeris based on non-singular elements. GPS Solut..

[17] Xiaogang X., Mingquan L. (2017). Broadcast Ephemeris Model of the BeiDou Navigation Satellite System. JESTR..

[18] Der G.J., Danchick R. Conversion of Osculating Orbital Elements to Mean Orbital Elements, Flight Dynamics. Proceedings of the Flight Mechanics/Estimation Theory Symposium 1996.

[19] Dong X., Hu C., Long T., Li Y. (2016). Numerical Analysis of Orbital Perturbation Effects on Inclined Geosynchronous SAR. Sensors.

[20] Montenbruck O., Steigenberger P. The BeiDou Navigation Message. Proceedings of the IGNSS Symposium 2013.

[21] Li H. (2014). Geostationary Satellites Collocation.

[22] Jiang M., Hu W., Ding C., Liu G. (2015). The effects of orbital perturbation on geosynchronous synthetic aperture radar imaging. IEEE Geosci. Remote Sens. Lett..

[23] Kaplan E.D., Leva J.L., Milbert D., Pavloff M.S., Kaplan E.D., Hegarty C.J. (2006). Global Positioning System. Understanding GPS—Principles and Applications.

[24] Xing Z., Wang X. (2012). Method of calculation numerical derivative of Jacobin matrix for fitting algorithm of GPS broadcast ephemeris parameters. GNSS World China.

[25] Xie X., Zeng D., Long T., Zang L. (2014). An improved ephemeris algorithm of Beidou GEO satellite for user. J. Nat. Uni. Defense Technol..

[26] Ouyang C., Shi J., Shen Y., Li L. (2019). Six-Year BDS-2 Broadcast Navigation Message Analysis from 2013 to 2018: Availability, Anomaly, and SIS UREs Assessment. Sensors.

[27] Montenbruck O., Steigenberger P., Hauschild A. (2015). Broadcast versus precise ephemerides: A multi-GNSS perspective. GPS Solut..

[28] Oh H., Park E., Lim H.-C., Park C. (2018). Orbit Determination of Korean GEO Satellite Using Single SLR Sensor. Sensors.

[29] Anghileri M., Paonni M., Fontanella D., Eissfeller B. (2013). Assessing GNSS Data Message Performance A New Approach. Inside GNSS.

[30] Xie W., Huang G., Cui B., Li P., Cao Y., Wang H., Chen Z., Shao B. (2019). Characteristics and Performance Evaluation of QZSS Onboard Satellite Clocks. Sensors.

[31] Chen H., Huang Y., Chiang K., Yang M., Rau R. (2009). The performance comparison between GPS and BeiDou-2/compass: A perspective from Asia. J. Chin. Inst. Eng..

